# A phase 2 trial of short-course Hyperthermic IntraPeritoneal Chemotherapy (HIPEC) at interval cytoreductive surgery (iCRS) for advanced ovarian cancer

**DOI:** 10.1590/0100-6991e-20223135

**Published:** 2022-02-18

**Authors:** THALES PAULO BATISTA, VANDRÉ CABRAL GOMES CARNEIRO, RODRIGO TANCREDI, LEVON BADIGLIAN, RONALDO LÚCIO COSTA RANGEL, ANDRÉ LOPES, BRUNO JOSÉ QUEIROZ SARMENTO, CRISTIANO SOUZA LEÃO

**Affiliations:** 1 - IMIP - Instituto de Medicina Integral Professor Fernando Figueira, Department of Surgery/Oncology - Recife - PE - Brasil; 2 - UFPE - Universidade Federal de Pernambuco, Center of Medical Science - Recife - PE - Brasil; 3 - HCP - Hospital de Câncer de Pernambuco, Department of Gynecology - Recife - PE - Brasil; 4 - IMIP - Instituto de Medicina Integral Professor Fernando Figueira, Department of Clinical Oncology - Recife - PE - Brasil; 5 - HCP - Hospital de Câncer de Pernambuco, Department of Clinical Oncology - Recife - PE - Brasil; 6 - AC Camargo Cancer Center, Department of Gynecology - São Paulo - SP - Brasil; 7 - IBCC - Instituto Brasileiro de Controle do Câncer, Department of Gynecology - São Paulo - SP - Brasil; 8 - IHBDF - Instituto Hospital de Base do Distrito Federal, Serviço de Oncologia Cirúrgica - Brasília - DF - Brasil; 9 - IMIP - Instituto de Medicina Integral Professor Fernando Figueira, Departamento de Cirurgia - Recife - PE - Brasil

**Keywords:** Neoadjuvant Therapy, Ovarian Neoplasms, Peritoneal Neoplasms, Terapia Neoadjuvante, Neoplasias Ovarianas, Neoplasias Peritoneais

## Abstract

**Objective:**

to report the final analysis of a phase 2 trial assessing the efficacy and safety of short-course hyperthermic intraperitoneal chemotherapy (HIPEC) for patients with advanced epithelial ovarian cancer (EOC).

**Methods:**

this was an open-label, multicenter, single-arm trial of HIPEC in patients with advanced EOC who underwent interval cytoreductive surgery (iCRS) after neoadjuvant chemotherapy (NACT). HIPEC was performed as a concentration-based regimen of platinum-based chemotherapy for 30 minutes. Primary endpoint was the rate of disease progression occurring at nine months following iCRS plus HIPEC (PD9). Secondary endpoints were postoperative complications, time to start adjuvant chemotherapy, length of hospital and ICU stay, quality of life (QoL) over treatment, and ultimately 2-year progression-free survival (PFS) and overall survival (OS). Analysis was by intention-to-treat with final database lock for survival outcomes on February 23, 2021.

**Results:**

fifteen patients with stage III EOC were enrolled between February 2015 and July 2019, in four centers. The intention to treat PD9 was 6.7%. With a median follow-up of 33 months (IQR, 24.3-46.5), the median PFS was 18.1 months and corresponding 2-year rates of PFS and OS was 33.3% and 93.3%, respectively. Three patients (20%) experienced graded III complications. Median length of hospital and ICU stay was 5 (IQR, 4-6.5) and 1 (IQR, 1-1) days, respectively. Time to restart systemic chemotherapy was 39 (IQR, 35-49.3) days and no significant difference over time in QoL was observed.

**Conclusions:**

we demonstrate preliminary efficacy and safety of short-course HIPEC in patient with advanced EOC.

## RESUMO

**Objetivo:**

apresentar a análise final de ensaio clínico de fase 2 que avaliou a eficácia e a segurança da quimioterapia intraperitoneal hipertérmica (HIPEC) de curta duração em pacientes com câncer epitelial de ovário avançado (EOC).

**Métodos:**

estudo aberto, multicêntrico, de braço único avaliando a HIPEC em pacientes com EOC avançado submetidos a cirurgia citorredutora de intervalo (iCRS) após quimioterapia neoadjuvante (NACT). A HIPEC foi realizada como regime baseado na concentração de cisplatina, perfundida por 30 minutos. O desfecho primário foi a taxa de progressão da doença 9 meses após a iCRS com HIPEC (PD9). Os desfechos secundários foram complicações pós-operatórias, tempo para iniciar a quimioterapia adjuvante, tempo de internação e permanência em UTI, qualidade de vida (QoL) ao longo do tratamento e, finalmente, sobrevida cumulativa livre de progressão (PSF) e global (OS) em 2 anos. As análises foram em intenção de tratar (ITT) com fechamento dos dados para análise da sobrevida em 23 de fevereiro de 2021.

**Resultados:**

quinze pacientes com EOC em estágio III foram incluídos no estudo entre fevereiro de 2015 e julho de 2019 em quatro centros recrutadores. A PD9 por ITT foi de 6,7%. Com acompanhamento mediano de 33 meses (IQR, 24,3-46,5), a PFS mediana foi de 18,1 meses e as taxas correspondentes de PFS e OS em 2 anos foram 33,3% e 93,3%, respectivamente. Três pacientes (20%) apresentaram complicações grau III. O tempo mediano de internamento hospitalar e em UTI foi de 5 (IQR, 4-6,5) e 1 (IQR, 1-1) dias, respectivamente. O tempo para reinício da quimioterapia sistêmica foi de 39 dias (IQR, 35-49,3) e não foi observada diferença significativa na QoL ao longo do tratamento.

**Conclusões:**

demonstrou-se eficácia e segurança preliminares da HIPEC de curta duração em pacientes com EOC avançado.

## INTRODUCTION

Ovarian cancer is a peritoneal-borne disease that tends to disseminate early into the peritoneal cavity and to be diagnosed at advanced stages in most patients. In these setting, hyperthermic intraperitoneal chemotherapy (HIPEC) has emerged as a main comprehensive treatment for patients with high tumor burden epithelial ovarian cancer (EOC) referred to neoadjuvant chemotherapy (NACT) because of poor clinical status or a low likelihood of achieving complete cytoreduction^1^. The rationale of HIPEC is based on the enhancement of cytotoxicity by hyperthermia for some anticancer medications and the pharmacokinetic advantages of intraperitoneal chemotherapy, whereas hyperthermia can also reduce the mechanisms of cellular resistance to platins and induce an efficient anticancer immune response via exposure of cell surface heat shock proteins^2^ ^-^ ^6^. This technique is finally delivered only during the operation, avoiding the need for implantation of peritoneal access devices and reducing catheter-related morbidity^7^. 

Unfortunately, there are a lot of worldwide variations of HIPEC procedures and several regimens of medications are available^8^, which have produced heterogeneous and no comparable results. However, recent published data have favored technical parameters such as higher concentration as well as higher temperatures for chemoperfusion^9^, suggesting the adoption of concentration-based regimens^10^ ^,^ ^11^ with intra-abdominal temperature of 41-43°C^12^ ^,^ ^13^ may be preferable to the body surface area-based regimen at 40°C applied in the landmark OVHIPEC trial^1^. Additionally, a shorter time of perfusion may also add benefits in terms of perioperative complications^14^.

We conducted this prospective trial to investigate the efficacy and safety of a short-course HIPEC protocol for the treatment of patients with advanced EOC. Herein, we report the results of this pioneering clinical trial, including perioperative complications and survival outcomes. 

## METHODS

### Study design and participants

This study was a non-randomized, open-label, multicenter, single-arm, phase 2 trial on the efficacy and safety of short-course HIPEC (i.e.: 30 minutes regimen) in advanced ovarian cancer. This regimen of HIPEC was explored into the context of a comprehensive strategy involving NACT, interval cytoreductive surgery (iCRS) plus short course HIPEC, and fast-track recovery procedures for patients with high tumor burden EOC. It was conducted under the hypothesis of low morbidity and improved outcomes for this all-in-one therapeutic approach that recruited patients from the Brazilian public health system named SUS - Sistema Único de Saúde.

Patients eligible for inclusion in the study were those with a biopsy-proven EOC who had at least stable disease and were refereed for interval cytoreduction after 2 to 4 cycles of NACT. Additional requirements for inclusion were aged 18-70 years; performance status of 0-2; clinical stage IIIB to IV limited to the abdomen; no synchronous malignancies or previous oncological treatments such as radiation or major abdominal operations; absence of neuro-psychiatric disorders, apparent or confirmed infections, history of medication allergies, and pregnancy or breast feeding; appropriated cardio-respiratory, hepato-renal and hematological reserves; and signing of the Consent Form. Exclusion criteria were having evidence of extensive retroperitoneal lymph node involvement or unresectable disease at the time of iCRS, limiting visceral obesity for the operation, and a residual disease after cytoreduction greater than or equal to 2.5mm (i.e.: CC-2 and CC-3).

The study protocol was reviewed by our Ethics Research Committees (Reference nº: CAAE 18388113.4.0000.5201, acceptance protocol at the coordinator center nº: 672.484; May 26, 2014) and thus registered on ClinicalTrials.gov under the identifier NCT02249013. Written informed consent was obtained from all patients and the procedures complied with the standards of current ethical guidelines. For safety monitoring, an interim analysis was also planned and previously reported^15^. Funding sources were from Decit/SCTIE/MS - CNPq/FACEPE/SES-PE (APQ:0187-4.01/13) and FAPE/IMIP.

### Procedures and Outcomes

Patients with advanced EOC treated with NACT due to high tumor load at diagnosis were referred to iCRS, and they were assessed to participate in this study if had at least stable disease up to 4 cycles of systemic chemotherapy. Systemic chemotherapy involves the total of 6 cycles of the standard combination of carboplatin (AUC 6) and paclitaxel (175mg/m^2^) administered every 21 days, adopting the usual criteria for dose modification or delay, as appropriated. Standard iCRS comprises maximal surgical efforts to reach no gross residual disease. Lymphadenectomy was at the surgeon’s discretion in patients with clinically suspicious of nodal involvement, and a more conservative policy of using high-voltage electrocoagulation, traditional scissor or knife resections, and other minor procedures were adopted as much as possible in order to reduce the morbidity, confining a complete peritonectomy to where there is evidence of a more bulky or confluent disease. A fast-track program based on “enhanced recovery after surgery” protocols (ERAS®, https://erassociety.org) was planned to accelerate recovery, reduce morbidity and shorten convalescence for patients enrolled in the trial, as previously reported^15^.

HIPEC was performed according to the closed-abdomen technique using cisplatin (25mg/L of perfusate/m^2^, total limit of 240mg) for the first 10 patients and thus, using cisplatin plus doxorubicin (15mg/L) thereafter, both for just 30 minutes, with an intra-abdominal target temperature of 41-43°C. Dianel perfusate was circulated using an extracorporeal circulation device named Performer HT (RanD, Medolla, Italy) at a flow rate of 700mL/min.

Primary endpoint was proportion of patients with disease progression or death at nine months following iCRS plus HIPEC (PD9) and secondary endpoints were complication rates according to the therapy-oriented Clavien-Dindo classification^16^, time to start adjuvant chemotherapy, length of hospital and ICU stay, quality of life (QoL) over treatment, and ultimately 2-year progression-free survival (PFS) and overall survival (OS). We defined PFS as the time from starting the NACT until the date of first progression or death, and OS as the time until death.

Clinical data on those patients enrolled into the trial were prospectively assessed and recorded by electronic spreadsheets. The follow-up scheduling for patient monitoring included clinical pelvic/general examination and CA125 every 3 months for two years, every six months for the next three years, then, annually. Imaging exams were also performed every 6-12 months or, when clinically required, for at least two years; and annually, thereafter.

Response to chemotherapy and progression were defined according to the comprehensive Gynecologic Cancer Intergroup (GCIG) criteria. At the time of operation, the peritoneal cancer index (PCI) and the completeness of cytoreduction (CC) scores were applied for the measurement of peritoneal spreading and classification for residual disease size, respectively. The extension of previous staging procedures was evaluated by means of the previous surgical score (PSS). Histological subtype and grade were assessed according to the usual World Health Organization (WHO) classification and surgical stage according to the International Federation of Gynecology and Obstetrics (FIGO) criteria as well. 

We measured the QoL using the European Organization for Research and Treatment of Cancer (EORTC) questionnaire QLQ-C30 v.3.0 (Brazilian Portuguese). This health-related QoL questionnaire was completed at baseline just before the iCRS plus HIPEC procedure (i.e.: at the time of hospital admission) and repeated after this comprehensive procedure (i.e.: at the time of re-stating the systemic chemotherapy) and after completion of the entire treatment protocol (i.e.: at 3-6 weeks after the last systemic chemotherapy cycle).

### Statistics

The study was initially designed to explore the efficacy of short-course HIPEC in terms of PFS as the primary outcome. However, due to slow accrual, the design was subsequently amended to explore the primary outcome measure of PD9, following publication of the OV21/PETROC trial^17^. Using an online tool for one-sample inference (https://www.stat.ubc.ca/~rollin/stats/ssize/b1.html), the sample size of 15 patients to be accrued was targeted in order to explore the initial relevance of our comprehensive treatment protocol with 80% power at one-sided 0.05 level, based on PD9 data extrapolated from OV21/PETROC trial (assumed reduction from 38 to 10%).

For descriptive analyses, we summarized continuous variables as medians (IQR, interquartile range) and categorical variables as frequencies (percent). Cumulative and median survival rates were estimated and plotted by the Kaplan-Meier method using the GraphPad PRISM Software version 9.0.1 (128). Analysis were by intention-to-treat with final database lock for survival outcomes on February 23, 2021. Changes of health-related QoL over time were assessed according to the EORTC QoL group procedures and analyzed by Friedman’s test at a significant two-sided level of 0.05.

### Role of the funding source

The decision to submit for publication was made after discussion with investigators of all the recruiting centers. The corresponding author and chief investigator (Batista TP) had full access to all the data in the study and had final responsibility for the decision to submit for publication. Funders had no influence on data analyses or decision for publishing.

## RESULTS

### Patients Characteristic

**Figure 1:**
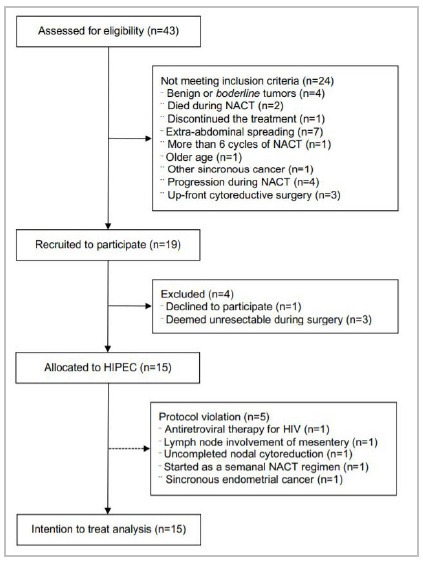
Patient assessment, recruitment, allocation and adherence to protocol (flowchart diagram).

Between February 2015 and July 2019, 43 women were assessed for eligibility (n=43) and 15 met the inclusion criteria and underwent HIPEC during iCRS in four of the six recruiting centers. Patients disposition throughout the study is shown as a flowchart diagram in Figure 1. One patient (n=1, 6.7%) staged IIIB at the exploratory laparotomy received NACT due to disease spreading into the upper abdomen and diffusing in the mesentery, while all other patients were selected as bulky stage IIIC disease (n=14, 93.3%). Baseline demographic and patient’s characteristics of patients are presented in Table 1.

**Table 1:** Baseline demographic and preoperative clinical characteristics.

Variable	Median (IQR) or n (%)
Age (years)	46 (42 - 59.5)
Body Mass Index	21.5 (19.5 - 24)
Performance Status (ECOG)^1^	
0	3 (20)
1	9 (60)
2	3 (20)
ASA Classification^2^	
I	9 (60)
II	6 (40)
Prior Surgical Score (PSS)	
0	7 (46.7)
1	7 (46.7)
2	1 (6.6)
FIGO Staging	
IIIB	1 (6.7)
IIIC	14 (93.3)
Histology	
High Grade Serous	11 (73.3)
Low Grade Serous	1 (6.7)
Endometrioid	2 (13.3)
Mixed Epithelial	1 (6.7)
BRCA Status (germline)	
BRCA1mut	2 (13.3)
BRCA2 VUS	2 (13.3)
BRCAwt	3 (20.1)
Not Assessed	8 (53.3)
Serum CA125 (U/mL) at Diagnosis	768.2 (522.5 - 1,397.6)
N° of cycles of Neoadjuvant Chemotherapy	3 (3 - 4)
N° of cycles of Adjuvant Chemotherapy	3 (2.3 - 3)

^*1*^ *Performance status at the time of iCRS plus HIPEC (after NACT).*

^*2*^ *ASA: American Society of Anesthesiologists Physical Status Classification.*

Footnotes: NACT: neoadjuvant chemotherapy; HIV: human immunodeficiency virus.

### Perioperative Outcomes and Adverse Events

As part of the iCRS, nine patients (60%) required bowel resection as rectosigmoidectomy (n=8) or partial colectomy (n=1), but no ostomies were performed. Most patients were extubated at the end of the operation (n=14, 93.3%) and left the ICU in the morning after the surgery (n=12, 80%). Systematic lymphadenectomies were not routinely performed in 8 of 15 patients (53.3%) while 7 of 15 underwent para-aortic lymph node dissection with (n=4) or without (n=3) pelvic lymphadenectomy. Twelve patients (80%) received no pelvic and/or abdominal drainage and in just 3 of 15 patients (20%) a chest tube was left. The vesical catheter was removed in the morning after surgery in 11 of 15 patients (73.3%). One patient (6.7%) receiving sodium thiosulphate during the operation to prevent nephrotoxicity, but no decline in kidney function was found in all the study population. Table 2 summarizes the operative characteristics and main postoperative outcomes. The corresponding body surface-area-based intraperitoneal dose of cisplatin was 125mg/m^2^ (IQR, 100.3-126) and dose modification (reduction) was not applied for any of patients.

**Table 2:** Operative and postoperative clinical characteristics.

Variables	Median (IQR) ou n (%)
Peritoneal Cancer Index (PCI) after NACT^1^	11 (8 - 15.5)
Complexity Score	
Low	2 (13.3)
Moderate	6 (40)
High	7 (46.7)
Variables	Median (IQR) ou n (%)
Completeness of Cytorreduction	
CC-0	14 (93.3)
CC-2^2^	1 (6.7)
Operative time (min)	490 (390 - 605.8)
Time of perfusion^3^ (min)	46 (44 - 51)
Mean temperature (°C)	42.1 (41.8 - 42.4)
Total dose of IP chemotherapy (mg)	
Cisplatin	180 (160 - 217.5)
Doxorrubicin	90 (75 - 90)
Hospital stay (days)	5 (4 - 6.5)
ICU stay (days)	1 (1 - 1)
Time to iCRS/HIPEC after NACT (days)	30 (27.5 - 32)
Time to Chemo after HIPEC (days)	39 (35 - 49.3)

^*1*^ *Peritoneal Cancer Index (PCI) at the time of iCRS plus HIPEC (after NACT).*

^*2*^ *Complete peritoneal cytoreduction with a residual bulky lymph node.*

^3^Total time after the “patient-filling phase”, while waiting for stables temperatures. The “drug circulation phase” (i.e.: HIPEC) was 30 minutes in all cases.

Three patients experienced no postoperative complications (20%), whereas 11 (73.3%) had minor grades I or II complications, and 3 (20%) presented grade III complications, according to the Clavien-Dindo classification. The total number of complications per patient was 1 (IQR, 1-2) and the most common complications were anemia (n=5) electrolytes imbalance (n=3), vomiting (n=3), lymphocele/lymph leakage (n=3). Two patients experienced reoperation, one due to postoperative hemorrhage and another for peritoneal infection; but no deaths were recorded. A summary of postoperative complications and adverse events is presented in Table 3. Two patients had long-term adverse events - one of them with encapsulating peritonitis as consequence of peritoneal infection without evidence of anastomotic leaks and the other one with brachial plexopathy related to the positioning during the long time of the surgical procedure. In both cases, patients recovered over time. 

**Table 3:** Postoperative complications ^1^ .

Variables	n (%)
Minor Complications (Grades I or II)	
Anemia	5
Electrolyte Imbalance	3
Vomiting	3
Abdominal distension	1
Bedsores	1
Brachial plexopathy	1
Catheter related infection	1
Constipation	1
Hypotension	1
Lymph leakage	1
Lymphocele	1
Wound infection	1
Major Complications (Grade III)	
Lymph leakage	1
Peritoneal infection	1
Postoperative hemorrhage	1

^1^Detailed description as total number of events, according to the therapy-oriented Clavien-Dindo classification.

One patient (6.6%) did not start adjuvant chemotherapy due to poor recovery after the operation; all others (n=14, 93.3%) completed six cycles of perioperative systemic chemotherapy as planned. Data on health-related QoL were previously reported^18^. In summary, no significant difference over time in the QLQ-C30 summary scores was observed (p>0.05).

### Pattern of Recurrences and Survival Outcomes

**Figure 2:**
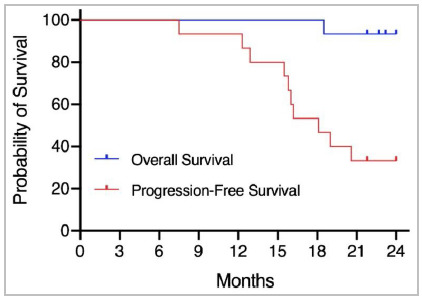
Kaplan-Meier estimates of PFS and OS for the intention-to-treat population. Median PFS was 18.1 months and median OS was not reached. The corresponding 2-year survival rates were 33.3% and 93.3%, respectively.

At the final database lock (February 23, 2021), 11 patients had a recurrent disease (73.3%) and six (40%) patients had died. The intention to treat PD9 was 6.7%. With a median follow-up of 33 months (IQR, 24.3-46.5), the median PFS was 18.1 months and the median OS was not reached. The corresponding 2-year survival rates were 33.3% and 93.3%, respectively. Kaplan-Meier estimates of PFS and OS for the intention-to-treat population is shown in Figure 2. 

All the relapses occurred in multiple locations such as nodal plus peritoneal involvement (n=4), extra-abdominal lymph nodes (n=2), nodal plus vaginal (n=2), extra-abdominal lymph nodes plus peritoneum (n=1), nodal, peritoneal and liver metastasis (n=1), and pleural plus osseous metastasis (n=1). After the first recurrence, three patients (20%) received secondary CRS (SeCRS) followed by systemic chemotherapy. Relapses in these cases were recorded at 16.2, 20.6 and 34.4 months. These patients underwent cardiophrenic lymph node resection through the diaphragmatic approach, peritoneal striping plus resection of pelvic lymph nodes plus splenectomy, and peritoneal removal plus debulking of mesenteric lymph nodes; respectively. One patient with low grade serous carcinoma received maintenance tamoxifen for 6 months after the adjuvant chemotherapy, but discontinued because of adverse events. At recurrence, this patient also received target therapy as bevacizumab plus systemic chemotherapy. No patients had access to treatment with PARP inhibitors and those not considered to SeCRS were treated with conventional systemic chemotherapy.

## DISCUSSION

HIPEC is a comprehensive treatment option for patients with advanced ovarian cancer. Using a body surface area-based escalating dose regimen of HIPEC perfused for 90 minutes at 40°C, the OV HIPEC trial by Van Driel et al.^1^ demonstrated HIPEC is cost-effective and improved both PFS and OS in patients advanced EOC^19^, with no major negatively impact in quality of life^20^. However, cytoreductive surgery (CRS) plus HIPEC protocols are widely variable and the best chemoperfusion regimen for EOC is still an open question^8^. In these settings, we present a short-course protocol as a promising alternative to the regimen proposed by the aforementioned landmark trial^1^. Our trial met its primary end-point and it can be well compared in terms of perioperative and survival outcomes with previous randomized controlled trials on NACT^21^ ^,^ ^22^, HIPEC^1^ and NACT plus intraperitoneal chemotherapy^17^, as well as with previous report on NACT from an experienced Brazilian center^23^. Based on our findings, this regimen is potentially effective and safe with the advantages in terms of postoperative outcomes such the short length of hospital stay, shorter time of chemoperfusion, and absence of any decline in kidney function. Of note, a marked advantage in terms of 2-year overall survival can be also noticed in this study. 

Classical data support a 30 minutes in-length regimen of hyperthermic chemoperfusion based on the activation of programmed cell death (apoptosis) by cisplatin^24^. Further, the systemic inflammatory response associated to prolonged exposure time during HIPEC might be the critical factor to increase its rates of postoperative infectious complications^14^ ^,^ ^25^. As reported by Roth et al.^14^, a secondary inflammatory reaction after 90 minutes of HIPEC that is usually associated to bacterial components in the systemic circulation is almost never observed after a short-course 30 minutes protocol^14^. Modern computational models for simulating the penetration of medication during HIPEC also point that moderate flow velocities, higher doses and higher temperatures are the most important factors to control the delivery of medication and to increase effectiveness of HIPEC procedures^9^. These findings are in accordance with recent experimental and clinical data supporting the adoption of concentration-based regimens instead of body surface area-based regimens of HIPEC^10^ ^,^ ^11^. Despite hyperthermia itself has not been proved to have direct cytotoxic effect on cancer cells at a therapeutic range of 41 to 43°C, platins display a temperature-dependent synergy with heat that increases medication uptake, DNA damage, and apoptosis at elevated temperatures. This synergism, however, requires temperatures higher than 41°C to increase effectivity^12^, whereas a temperature threshold above of 40°C is also critical to improve survival outcomes in patients undergoing HIPEC for peritoneal malignancies^13^. All of these concerns are present in our short-course regimen of HIPEC and can explain the favorable outcomes in terms survival outcomes.

Biological characteristics of EOC are favorable to the use of HIPEC in the specific time-point of interval cytoreduction, which is supported by a landmark phase 3 trial^1^ and by the long-term survival advantages of intraperitoneal chemotherapy^26^. Accordingly, patients requiring neoadjuvant chemotherapy due to extensive disease and/or poor clinical status at diagnosis are probably those who benefit the most of HIPEC because the need of treatment intensification to compensate the detrimental influence of the tumor burden either at the diagnosis^21^ ^,^ ^22^ ^,^ ^27^ or at the time of iCRS^28^, and because of the higher risk of developing platinum resistance after NACT^23^. In these settings, HIPEC may serve to reverse or circumvent the resistance to platinum-based chemotherapy via inhibition of homologous recombination mechanisms^29^, activation of heat-shock proteins that are able to modify multiple cellular functions^3^ ^,^ ^5^ ^,^ ^6^, and epigenetic alterations^30^, which ultimately may induce sensibility to PARP inhibitors even in innately homologous recombination-proficient patients^29^ ^,^ ^31^. These translational and preclinical data are supported by clinical studies that demonstrated similar survival in both platinum-sensitive and platinum-resistant disease in patients who underwent HIPEC^2^ ^,^ ^4^, and it may explain the almost one year benefit in terms of overall survival observed in the OVHIPEC trial^1^. A trend of this benefit can also be noticed in the current study.

Interestingly, early tumor regrowth has been observed after complete cytoreduction in about ¼ of patients at the time of postoperative radiological assessment for starting adjuvant chemotherapy^32^. Similarly, many patients have microscopic disease on pathological assessment when no disease is macroscopically evident at iCRS, even after a median of five cycles of NACT^33^. In a mathematical model designed to determine the probability of presenting microscopic peritoneal metastases after CRS, the probability of residual disease after complete cytoreduction was estimated by 98.14% in patients with EOC^34^. This clearly demonstrates the need for earlier initiation of adjuvant treatments after CRS in patients with advanced ovarian cancer, and HIPEC represents the best comprehensive approach to target these issues. Further, HIPEC may also add immune-related benefits able to improve OS in patients with advanced EOC^35^.

This study has several limitations. Mainly, it was limited by the slow accrual that had lead us to review our study design and to anticipate the completion of the trial. Although the efforts of other Brazilian cancer centers to participate in the trial, we experienced the difficulties for developing surgical trials that required major changes in surgical practices. Additional criticisms to our protocol are that the starting protocol lacked to provide a routine laparoscopic estimation of tumour burden at the diagnosis, BRCA assessment was not possible for all patients, a baseline QoL measurement just before starting NACT was not required, and a high rate of protocol violations. Another main point of interest was the use of two different regimens of intraperitoneal chemotherapy during HIPEC. Despite a doublet regimen being potentially a better regimen for HIPEC, we had initially planned to start with a single regimen in order to simplify our procedures in the first 10 cases. Nowadays, we believe that a doublet regimen is especially important to improve outcomes of HIPEC after neoadjuvant chemotherapy, probably modifying the response of remaining neoplastic cells to systematic chemotherapy^2^. However, due to the small number of cases included in the study, a separate analysis of the two applied regimens of medications is not feasible. 

On the other hand, the strengths of this study include the fact it was the first clinical trial involving HIPEC procedures and the first to use the Performer HT device (RanD, Mendoll, IT) in Brazil. This includes the efforts of conducting such type of study into the context of the public health system in a developing country. We can also highlight the pioneering of exploring a comprehensive strategy combining perioperative chemotherapy, advanced cytoreductive techniques, fast-track recovery procedures and short-course HIPEC for patients suffering of advanced EOC. Our short-course HIPEC regimen also sums most of the recent concerns deemed important to improve outcomes after HIPEC, despite having been planned between 2013 and 2014. Finally, our comprehensive protocol appears very feasible to be used in the real-world settings. 

In conclusion, we confirm the preliminary hypothesis of efficacy and safety of our comprehensive approach involving the use of short-course HIPEC in advanced EOC. This regimen might be an alternative to previous published regimens and deserves further evaluation in randomized controlled trials. A Bayesian pick-the-winner design or a proof-of-concept non-inferiority criterion (i.e.: supposing at least non-inferiority plus lower morbidity) for a subsequent randomized phase 2 clinical trial comparing these regimens may quickly clarify whether a short-course regimen should be preferred.
